# In Depth Analysis on the Binding Sites of Adamantane Derivatives in HCV (Hepatitis C Virus) p7 Channel Based on the NMR Structure

**DOI:** 10.1371/journal.pone.0093613

**Published:** 2014-04-08

**Authors:** Qi-Shi Du, Shu-Qing Wang, Dong Chen, Jian-Zong Meng, Ri-Bo Huang

**Affiliations:** 1 State Key Laboratory of Non-food Biomass and Enzyme Technology, National Engineering Research Center for Non-food Biorefinery, Guangxi Academy of Sciences, Nanning, Guangxi, China; 2 Tianjin Key Laboratory on Technologies Enabling Development of Clinical Therapeutics and Diagnostics (Theranostics), School of Pharmacy, Tianjin Medical University, Tianjin, China; 3 Life Science and Biotechnology College, Guangxi University, Nanning, Guangxi, China; 4 Gordon Life Science Institute, San Diego, California, United States of America; University of Washington, United States of America

## Abstract

**Background:**

The recently solved solution structure of HCV (hepatitis C virus) p7 ion channel provides a solid structure basis for drug design against HCV infection. In the p7 channel the ligand amantadine (or rimantadine) was determined in a hydrophobic pocket. However the pharmocophore (−NH_2_) of the ligand was not assigned a specific binding site.

**Results:**

The possible binding sites for amino group of adamantane derivatives is studied based on the NMR structure of p7 channel using QM calculation and molecular modeling. In the hydrophobic cavity and nearby three possible binding sites are proposed: His17, Phe20, and Trp21. The ligand binding energies at the three binding sites are studied using high level QM method CCSD(T)/6–311+G(d,p) and AutoDock calculations, and the interaction details are analyzed. The potential application of the binding sites for rational inhibitor design are discussed.

**Conclusions:**

Some useful viewpoints are concluded as follows. (1) The amino group (−NH_2_) of adamantane derivatives is protonated (−NH_3_^+^), and the positively charged cation may form cation-π interactions with aromatic amino acids. (2) The aromatic amino acids (His17, Phe20, and Trp21) are the possible binding sites for the protonated amino group (−NH_3_^+^) of adamantane derivatives, and the cation-π bond energies are 3 to 5 times stronger than the energies of common hydrogen bonds. (3) The higher inhibition potent of rimantadine than amantadine probably because of its higher pK_a_ value (pK_a_ = 10.40) and the higher positive charge in the amino group. The potential application of p7 channel structure for inhibitor design is discussed.

## Introduction

Hepatitis C is an infectious disease [1] caused by the hepatitis C virus (HCV) [2], primarily affecting the liver. Hepatitis C is the leading cause for liver diseases in the USA. About 200 million people are infected with HCV worldwide [3]. The chronic infection of HCV can lead to scarring of the liver and ultimately to cirrhosis, which is generally apparent after many years. In some cases, those with cirrhosis will go on to develop liver failure, liver cancer or life-threatening esophageal and gastric varices [1]. So far there is no clinically proven vaccine [4], [5], and the most common therapy is based on a combination therapy of pegylated interferon-alpha (PEG-IFNa) and ribavirin (RBV), which only has a success rate of around 50% as well as severe side effects [6], [7]. Development of more effective new drugs is absolutely necessary.

The p7 channel plays multiple roles in virus life cycle and has several biological functions in HCV infection. Therefore the HCV p7 protein has been sought after as a potential anti-HCV drug target [8], [9]. The p7 is a 63-residue membrane protein that oligomerizes to form ion channels with cation selectivity, for Na^+^, K^+^, and Ca^2+^ [10]–[13], and a more recent study has reported that the p7 channel mediated H^+^ intracellular conductance [14]. The adamantane derivatives and other several compounds [10]–[15] have been used in HCV clinical trials, but large variation in drug efficacy among the various HCV genotypes has been difficult to explain, because the drug target structure information of adamantane derivatives was not available, and the drug-target interaction mechanism was not clear.

**Figure 1:**
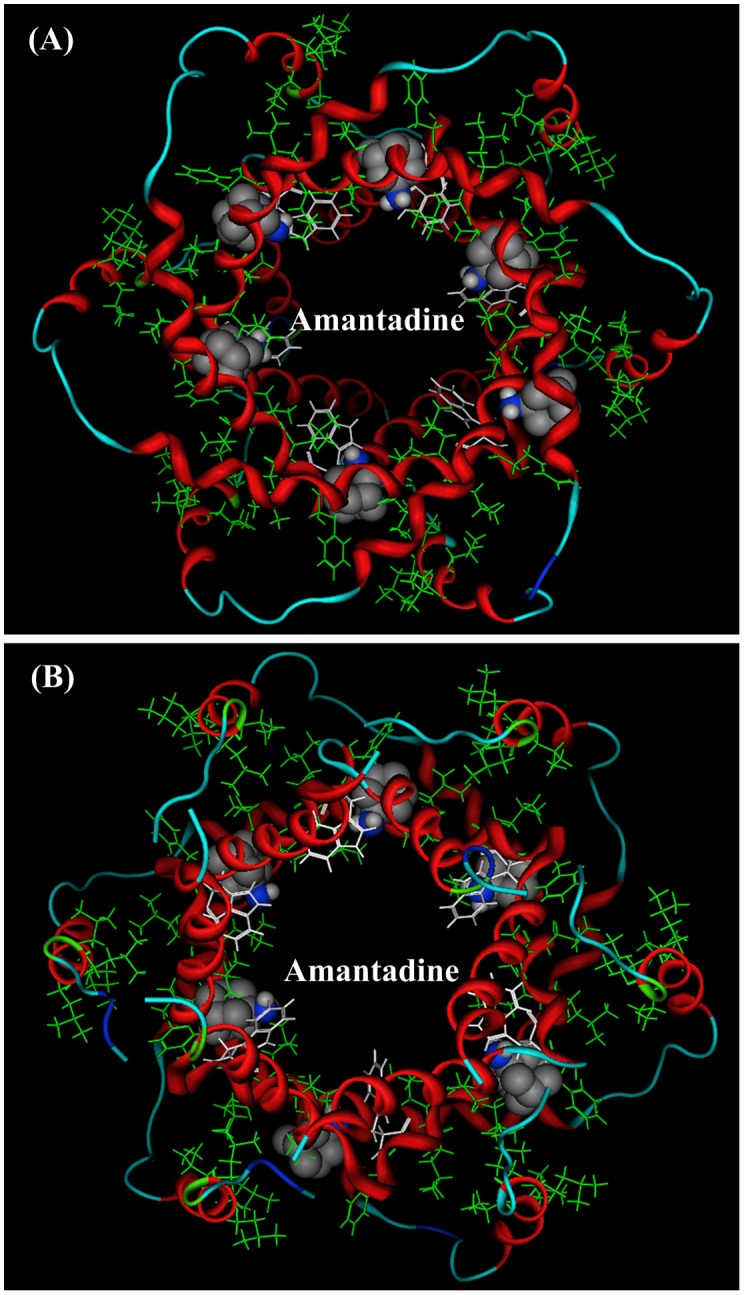
NMR solution structure (PDB code: 2M6X) of HCV p7 ion channel and the positions of ligand amantadine. In p7 ion channel there are six equivalent hydrophobic pockets between the peripheral and pore-forming helices. The ligand amantadine (or rimantadine) is located in the hydrophobic cavities. The pocket consists of Phe 20, Val 25, Val26, Leu52, Val53, Leu55, and Leu56,. The amino group of amantadine on average points to the channel lumen [16]. (**A**) A view from the top of channel. (**B**) A view from the bottom of channel.

**Figure 2:**
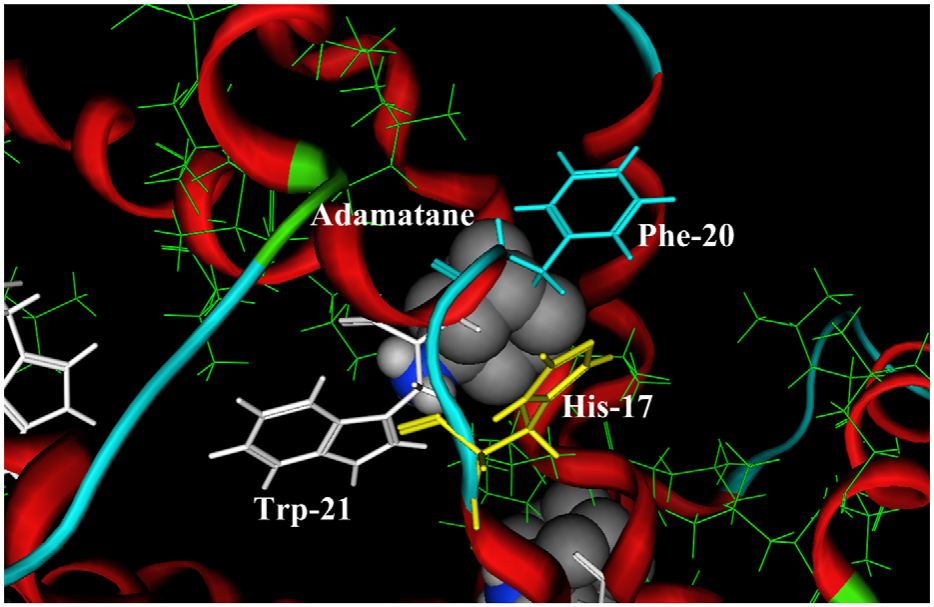
A close view of the binding pocket of amantadine in the p7 ion channel. The hydrophobic residues (Phe20, Val25, Val26, Leu52, Val53, Leu55, and Leu56) are shown in green line drawing, which comprise the hydrophobic binding pocket of amantadine. The positions of three possible binding sites His17, Phe20, and Trp21 for the protonated pharmocophore group (−NH_3_^+^) of amantadine are shown in yellow, light blue, and white, respectively. All three aromatic residues (His17, Phe20, and Trp21) are on the chain 2.

Recently the p7 channel structure in solution was solved by Chou and colleagues [16] using the state of the art NMR techniques. The top view and bottom view of the p7 channel are shown in **Fig. 1** (**A**) and (**B**), respectively. In the p7 channel there are six equivalent hydrophobic pockets between the peripheral and pore-forming helices, consisting of Leu 52, Val 53 Leu55 and Leu 56 from H3, and Phe 20, Val 25 and Val 26 from H2 [16]. In **Fig. 1** these hydrophobic residues are shown in green lines. The ligand amantadine (or rimantadine) is located in the hydrophobic cavities. **Fig. 2** is a close-up view of the binding location of ligand amantadine in the p7 ion channel, which is drawn based on the description in ref [16]. The binding location of amantadine (or rimantadine) in p7 ion channel, identified by Chou’s lab, is different from the binding mode in the influenza M2 channel [17]–[19]. It turns out to be crucial to the functioning of the drug mechanism. While the tiny M2 channel in influenza gets plugged up by the drug molecule [18], in the p7 channel the drug nestles into a series of pockets within a folded outside edge of the funnel. When the drugs are in those pockets, the channel is unable to “exhale” and thus release ions [16].

In Chou’s binding model of the p7 ion channel the amantadine (or rimantadine) is located in the hydrophobic cavity comprised by Phe20, val25, val26, Leu52, Val53, Leu55, and Leu56. The hydrophobic adamantane body of amantadine is surrounded by above hydrophobic amino acids in the cavity. However, the binding site of the adamantane’s pharmocophore (−NH_2_) in the p7 ion channel was not accurately reported in ref [16], as saying the amino group (−NH_2_) of amantadine “*on average points to the channel lumen*” [16].

A pharmacophore is an abstract description of molecular features of a drug family, which are necessary for molecular recognition of a ligand by a biological macromolecule. Usually the drug pharmocophore makes the main contribution to the binding free energy and plays the key role in inhibition activity. The IUPAC defines a pharmacophore to be “*an ensemble of steric and electronic features that is necessary to ensure the optimal supramolecular interactions with a specific biological target and to trigger (or block) its biological response”* [39]. In the M2 proton channel [17]–[19] of influenza A virus the ligand adamantane derivatives may bind at more than one position, or the ligand binding location may change at deferent biological stages [20].

The authors of ref [16] pointed “*the relatively poor stability of the protein–drug complex at the current stage of our study precludes full-scale structure determination*”. In this situation molecule modeling and accurate QM calculation may help to solve the binding sites of the pharmocophore group (−NH_2_) within the hydrophobic pocket of p7 ion channel.

## Method and Theory

Adamantane derivatives are alkaline compounds. The pK_a_ values of residues are important for binding interactions. In proteins the pK_a_ values of residues can change in a broad range because of the influence from the interaction environment. The pK_a_ value of an ionizable amino acid is evaluated using the following equation,
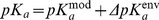
(1)In **Eq. 1** the pK_a_^mod^ is a model value assigned to certain amino acid types. For histidine the model value pK_a_^mod^ = 6.50. The term ΔpK_a_^env^ is the correction value from the environment residues in protein. The acidic dissociation constants pK_a_ values of all acidic and alkaline residues in p7 ion channel are calculated using PROPKA3.1 software package [21]–[24].

The binding free energies between ligand and host protein at three possible binding sites (His17, Phe20, and Trp21) are calculated by using AutoDock 4.0 software package [25], and the grid maps of the protein used for docking process were calculated with the AutoGrid 4.0 [26]. The grid dimensions centered at the defined pocket were 40×40×40 grid points with a spacing of 0.375 Å in each dimension. Gasteiger charges, computed by ADT (AutoDock tools), were assigned to both amantadine and receptor. Docking simulations were performed with the Lamarckian Genetic Algorithm [25]–[27], using maximum number of 250,000 energy evaluations, mutation rate of 0.02, cross over rate of 0.08, and elitism value of 1. All other docking parameters were left at the default values. Each docking job included 200 independent runs. Finally, the docked poses for each ligand within 1.0 Å in the root mean square deviation (rmsd) tolerance of each other were clustered together with the really close binding energy. In the calculations a flexible model is used for both ligand and host acceptor. It means that the ligand and host protein can adjust their conformations and orientation to make the best docking effect.

The protonated amino group (−NH_3_^+^) of amantadine (or rimantadine) can forms a stable cation-π bond with the aromatic residues. The physical nature and properties of cation-π interactions [28]–[32] are very different from the well known three type interactions: van der Waals interaction, electrostatic interaction, and hydrogen bond interaction. Some available force field parameters may not include the cation-π interactions correctly. In cation-π interactions the electron correlation and dispersion interaction make important contribution, which have to be described by using post Hartree-Fock methods, such as MP2, CCDS or CCSD(T) [33]–[36]. In this study the structures of molecular monomers are optimized using CCSD/6–311+G(d,p), and the cation-π interaction energies are calculated at CCSD(T)/6–311+G(d,p) level.

## Results

In this section the computational and modeling results are reported using tables and figures, and followed by brief illustration and comparison.

### Acidic Dissociation Constants (pK_a_) of Residues

The pharmocophore (−NH_2_) of rimantadine and amantadine are alkaline group that is sensitive to the acidic dissociation constants (pK_a_) values of amino acids. The pK_a_ values of residues in proteins can change in a broad range because of the influence from the interaction environment. The pK_a_ values of residues in p7 ion channel are calculated by using the PROPKA3.1 software [22]–[24], [37], and the results are listed in **Table 1**.

**Table 1:** The calculated *pK_a_ values of amino acids in p7 protein.

A. A.	Position	Chain	pKa	A. A.	Position	Chain	pKa
HIS	17	A	6.07	LYS	3	D	10.30
HIS	31	A	5.44	LYS	33	D	9.58
HIS	59	A	5.77	LYS	3	E	10.05
HIS	17	B	5.52	LYS	33	E	9.60
HIS	31	B	5.63	LYS	3	F	10.13
HIS	59	B	5.56	LYS	33	F	9.51
HIS	17	C	6.12	ARG	35	A	11.95
HIS	31	C	5.62	ARG	54	A	12.37
HIS	59	C	5.81	ARG	57	A	12.34
HIS	17	D	5.89	ARG	60	A	12.42
HIS	31	D	5.56	ARG	35	B	12.07
HIS	59	D	5.73	ARG	54	B	12.35
HIS	17	E	6.22	ARG	57	B	12.27
HIS	31	E	5.63	ARG	60	B	12.37
HIS	59	E	5.77	ARG	35	C	12.10
HIS	17	F	5.53	ARG	54	C	12.33
HIS	31	F	5.50	ARG	57	C	12.30
HIS	59	F	5.87	ARG	60	C	12.44
TYR	42	A	9.94	ARG	35	D	12.06
TYR	42	B	10.03	ARG	54	D	12.38
TYR	42	C	10.21	ARG	57	D	12.31
TYR	42	D	10.13	ARG	60	D	12.44
TYR	42	E	10.38	ARG	35	E	12.03
TYR	42	F	10.29	ARG	54	E	12.33
LYS	3	A	10.13	ARG	57	E	12.27
LYS	33	A	9.12	ARG	60	E	12.43
LYS	3	B	10.10	ARG	35	F	11.96
LYS	33	B	9.88	ARG	54	F	12.33
LYS	3	C	10.20	ARG	57	F	12.20
LYS	33	C	10.01	ARG	60	F	12.44
Amantadine		9.00 [28]		Rimantadine		10.40 [28]	

*PROPKA3.1 [24]–[27]. (http://propka.ki.ku.dk/pka/2M6X.html).

Among the 378 amino acids (63×6) only 18 histidine residues (His17, His37, His59) are acidic, and the pK_a_ values of histidine residues are in the region 5.44 to 6.22. On the other hand, 42 residues (Lys3, Lys33, Trp42, Arg35, Arg54, Arg57, and Arg60) are alkaline amino acids. Among them the pK_a_ values of 24 arginine residues are larger than pK_a_>12. The p7 cation channel is an alkaline amino acid dominated protein.

In the last two lines of **Table 1** the pK_a_ values of amantadine (pK_a_ = 9.00) and rimantadine (pK_a_ = 10.40) are checked from ref [38]. In common cell condition most molecules of amantadine and rimantadine appear in protonated form (Ad−NH_3_^+^).

### Possible Binding Sites for Amantadine Pharmocophore (−NH^+^_3_)

As described in ref [16], the binding location of amantadine (or rimantadine) in p7 ion channel is in the hydrophobic pocket comprised by Phe20, val25, val26, Leu52, Val53, Leu55, and Leu56. This result is overall consistent with a mutational study [38] showing that mutations in residues 50–55 significantly reduce drug sensitivity of the channel.

The pharmocophore (−NH^+^_3_) of amantadine is an alkaline group, also a cation. The possible binding interactions are acid-alkaline interaction and cation-π interaction. In the hydrophobic pocket and nearby there are three aromatic amino acids (His17, Phe20, and Trp21). The His17 is also an acidic residue. The three residues are the possible binding sites for the protonated amino group (−NH_3_^+^) of amantadine and rimantadine. The positions of three residues (His17, Phe20, and Trp21) are shown in **Fig. 2**. All the three residues are on the chain 2.

#### (1) Binding site His17

**Figure 3:**
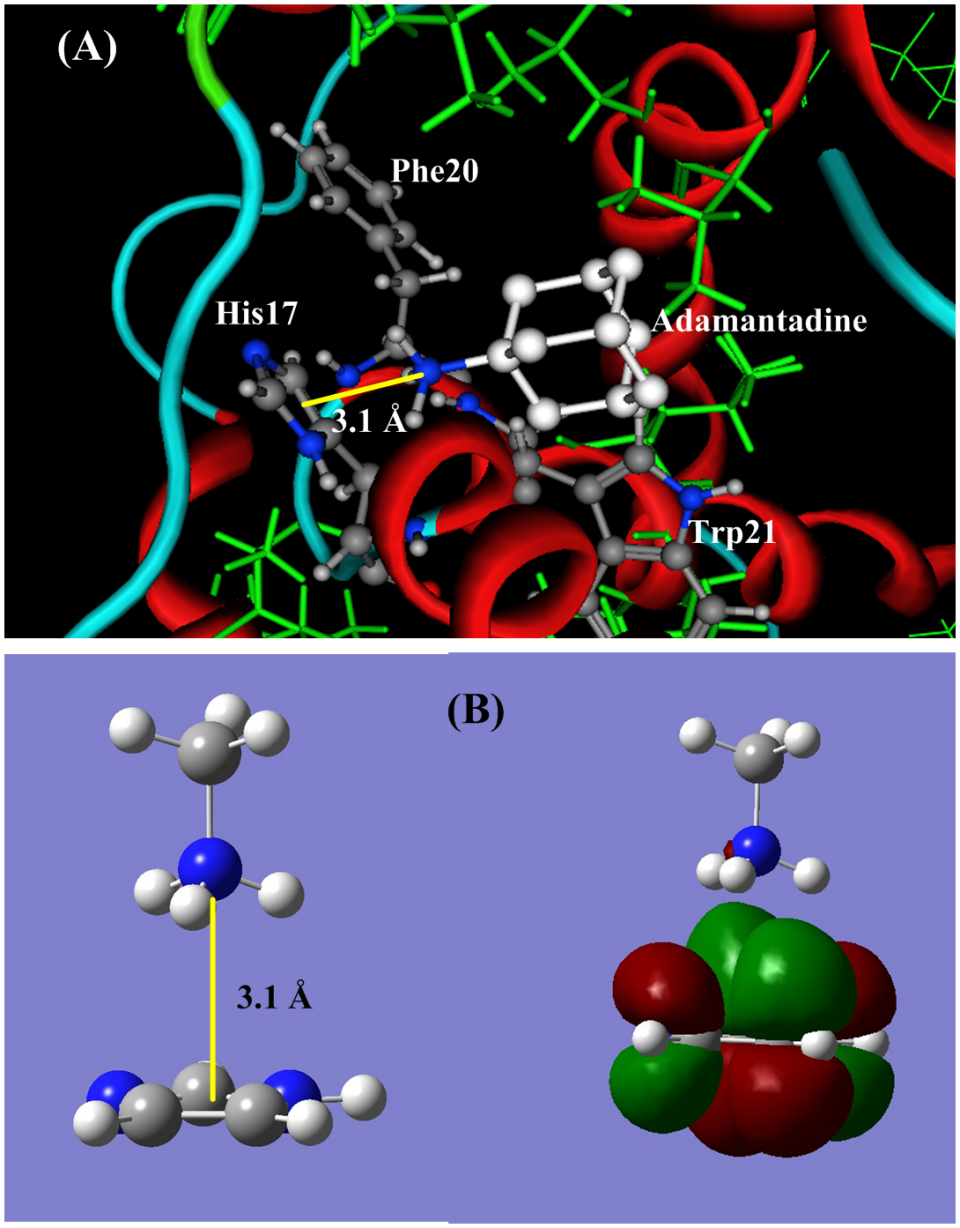
The ligand-acceptor binding interaction between amantadine and p7 ion channel at binding site His17. (**A**) The docking structure between amantadine and p7 channel at the site His17. (**B**). QM calculation for cation-π interaction between CH_3_NH_3_^+^ and the aromatic side chain of His. The protonated amino group (−NH_3_^+^) of amantadine perpendicularly points to the π-plane of His17, and forms a stable cation-π bond. The cation-π bond length is 3.072 Å and the interaction energy is −50.28 kJ/mol.

Histidine is the only acidic amino acid in p7 protein, possessing the pK_a_ = 5.44∼6.22 in p7 channel, little lower than 7. In the common cell condition histidine may appear in either protonated form or neutral form. When the residue His17 is in neural form, the protonated amino group (−NH_3_^+^) of amantadine (or rimantadine) can forms a stable cation-π bond [28]–[32] with the aromatic side chain imidazole of His17. A docking structure of cation-π interaction between amantadine and His17 is shown in **Fig. 3** (**A**), and **Fig. 3** (**B**) is an illustration portrait for the cation-π interaction between CH_3_NH_3_^+^ and imidazole (side chain of His17), in which the protonated amino group (−NH_3_^+^) perpendicularly point to the π-plane of imidazole ring. The distance from nitrogen atom to the π-plane is 3.1 Å, and the bond energy is −50.28 kJ/mol, 2.5 times of the common hydrogen bond energies (∼−20 kJ/mol).

Among the 20 natural amino acids histidine is the unique member that can join 5 type molecular interactions (hydrogen bond, cation-π, polar hydrogen-π, π–π stocking, and coordinate interaction) [28]. The pK_a_ value of histidine can change in a broad range around 6. 0, affected by the interaction condition. Consequently histidine could be a proton donor or acceptor, and appear in neutral form or protonated form. In the p7 ion channel the His17 residues may play an important role in the cation conductance. Therefore the binding of amantadine on the His17 may significantly affect the biological function of p7 ion channel.

#### (2) Binding site Phe20

**Figure 4:**
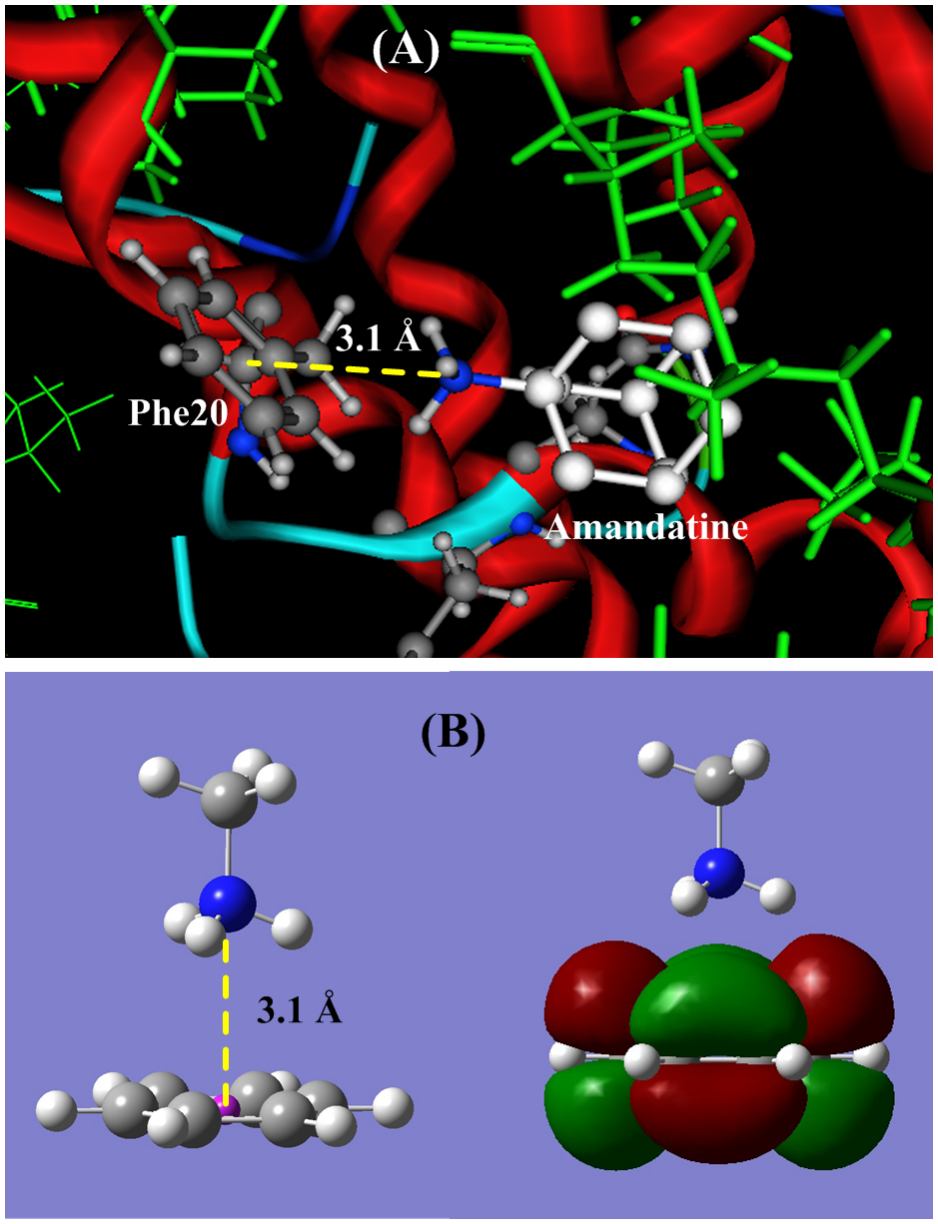
The ligand-acceptor binding interaction between amantadine and p7 channel at binding site Phe20. (**A**) The docking structure between amantadine and p7 channel at the site Phe20. (**B**). QM calculation for cation-π interaction between CH_3_NH_3_^+^ and the aromatic side chain of Phe. The protonated amino group (−NH_3_^+^) of amantadine perpendicularly points to the π-plane of Phe20, and forms a stable cation-π bond. The cation-π bond length is 3.084 Å and the interaction energy is −62.80 kJ/mol.

In the p7 ion channel [16] the Phe20 is an artificial mutation that replaced the Leu20 in wild HCV p7 channel. The aromatic amino acid Phe20 is a part of the hydrophobic pocket and directly contacts with the ligand amantadine or rimantadine [16], as shown in **Fig. 2** (**A**)**.** The distance between Phe20 and amino group of amantadine is 3.6 to 4.0 Å, in the cation-π interaction region. **Fig. 4** (**A**) shows the docking structure between amantadine and Phe20, in which the protonated amino group (−NH_3_^+^) perpendicularly points to the π-plane of Phe20, and a stable cation-π bond is formed. **Fig. 4** (**B**) is an illustration portrait for the cation-π interaction between CH_3_NH_3_^+^ and C_6_H_6_ (side chain of Phe20). The optimized distance from nitrogen atom to the π-plane is 3.1 Å, and the cation-π interaction energy is −62.80 kJ/mol, three folds as the common hydrogen bond energy.

#### (3) Binding site Trp21

**Figure 5:**
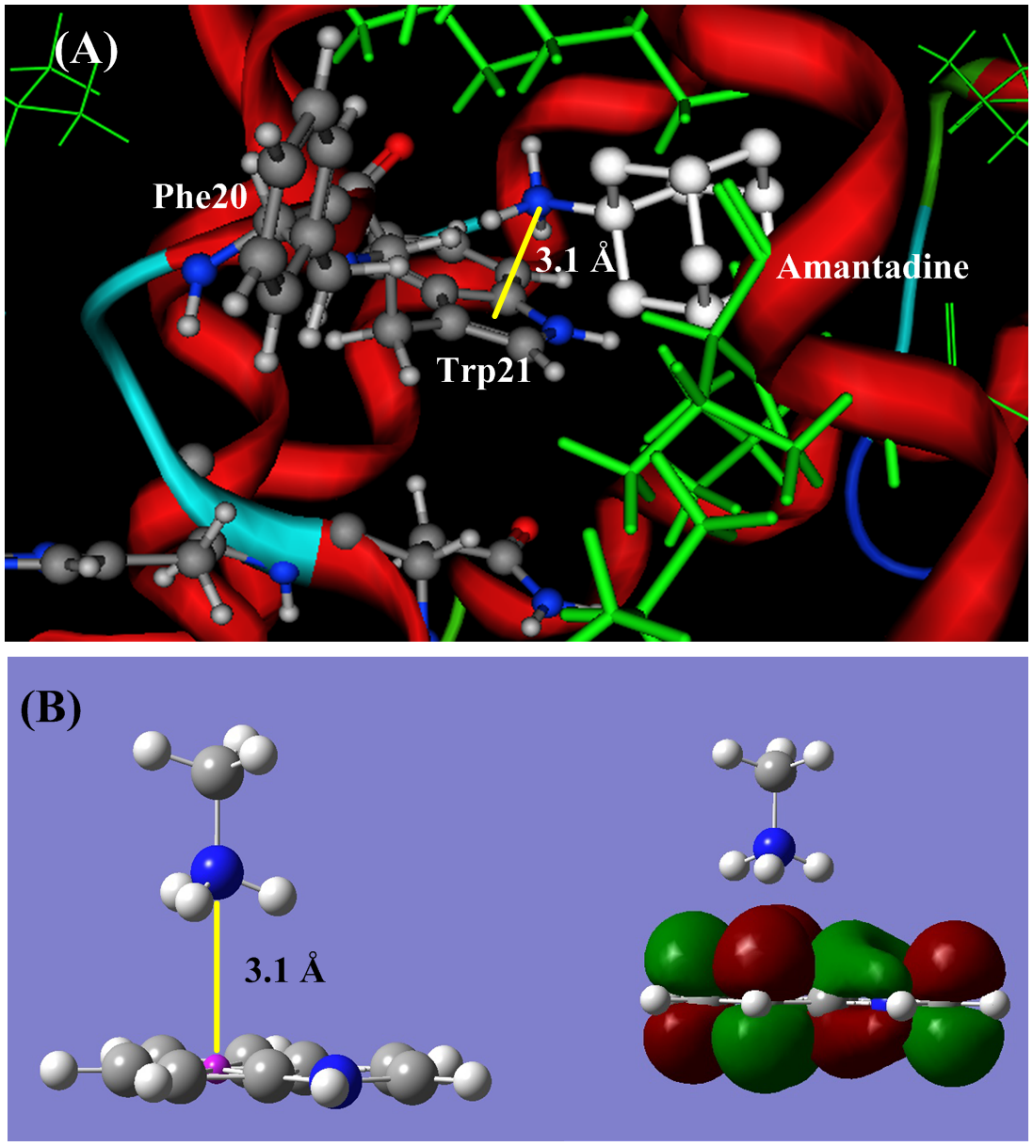
The ligand-acceptor binding interaction between amantadine and p7 channel at binding site Trp21. (**A**) The docking structure between amantadine and p7 channel at the site Trp21. (**B**). QM calculation for cation-π interaction between CH_3_NH_3_^+^ and the aromatic side chain of Trp. The protonated amino group (−NH_3_^+^) of amantadine perpendicularly points to the π-plane of Trp21, and forms a stable cation-π bond. The cation-π bond length is 2.99 Å and the interaction energy is −82.53 kJ/mol.

The tryptophan is the largest aromatic amino acid in the 20 natural amino acids. In the p7 channel the positions of Trp21 and Phe20 are on the two sides of the ligand amantadine: Trp21 is close the amino group, and Phe20 is near the adamantine body of the amantadine. The side chain of Trp21 plugs into the p7 channel. Similar to the Phe20, the aromatic amino acid Trp21 can form cation-π interaction with the protonated amino group (−NH_3_^+^) of amantadine (or rimantadine). The binding interaction structure of amantadine-Trp21 is shown in **Fig. 5** (**A**). The cation-π interaction energy (−82.53 kJ/mol) between Trp21 and amantadine is four folds as the common hydrogen bond energy, which is the largest one among the three aromatic amino acids because of its large aromatic π-system.

### Calculations of Binding Free Energies

In the above calculations the binding energies between amantadine and three aromatic amino acids are calculated by using QM method. The binding energies are the main contribution to the binding free energies. However, the full binding free energies have to be calculated using docking method. The docking free energies between ligands amantadine and p7 channel at the three binding sites (His17, Phe20, and Trp21) are calculated using AutoDock4.0 [25]. In the calculations a flexible model [26], [27] for both ligand and host acceptor is used, meaning that the local conformations of both ligand and host protein can be adjusted to reach the best docking effect. The calculated docking free energies are listed in **Table 2**.

**Table 2:** Binding free energies and cation-π interaction energies of amantadine (or rimantidine) at three possible binding sites (His17, Phe20, and Trp21) of p7 ion channel.

*Docking calculation:			
Binding sites	His17	Phe20	Trp21
Free energy	−19.09	−22.81	−24.53
** **CCSD(T)/6–311+G(d,p) calculation:**			
Interaction pair	CH_3_NH_3_^+^−His	CH_3_NH_3_^+^−Phe	CH_3_NH_3_^+^−Trp
Cation-π energy	−50.278	−62.80	−82.53

*Calculated using Autodock4.0 [33], energy in kJ/mol.

**CCSD/6–311G(d,p) [37]–[40], energy in kJ/mol. In QM calculations the protonated amantadine is simplified as CH_3_NH_3_^+^, and the aromatic amino acids are replace by their aromatic side chains.

In **Table 2** the binding free energies of docking calculations are around −20 kJ/mol, much smaller than the cation-π interaction energies that are calculated using high level quantum chemical method CCSD(T)/6–311+G(d,p) [33]–[36]. The binding free energies include all interaction terms between ligand and host protein. The cation-π interaction energy is the main contribution to the binding free energy. In the docking calculations the binding free energies depend on the force field parameters [30], [40]–[43]. However, the cation-π interaction energies may be not correctly described by the available force field parameters [30], [40]–[43]. Despite the value differences between binding free energies (dock calculations) and cation-π energies (QM calculations), the order of the two calculation methods are the same: Trp21>Phe20>His17. As shown in **Table 2**, the energies of cation-π interactions are much larger (2.5 to 4 folds) than the energies (∼−20 kJ/mol) of common hydrogen bonds.

## Discussion

In this study the binding-site searching for pharmocophore group (−NH^+^_3_) of adamantane derivatives are performed in the hydrophobic binding pocket comprised by Phe20, val25, val26, Leu52, Val53, Leu55, and Leu56, which is determined by NMR experiment [16], and supported by the previous mutation experiments [15], [38], [46]. It is common that the ligands may have more than one biding sites [44] in the host proteins, and the ligand binding sites may change at different biological stages [45]. Within the binding pocket of adamantane derivatives, three possible binding sites (His17, Phe20, and Trp21) for the amino group of amantadine (or rimantadine) are suggested based on high level QM calculations and docking calculations.

In the binding energy calculations we pay high attention on the acid-alkaline interaction and cation-π interaction, because the amantadine derivatives are alkaline compounds, and also cations. The cation-π interaction energies are much stronger than van der Waals interactions, electrostatic interactions, and common hydrogen bonding interactions. The cation-π interaction energies (**Table 2**) between amantadine and aromatic amino acids are 2.5 to 4 folds as the common H_2_O-H_2_O hydrogen bond energy (∼−20 kJ/mol). The cation-π interactions are the interactions between cation group and π-plane in ‘T’ form, having broader interaction space and longer interaction distance than the hydrogen bonds.

Among the three aromatic amino acids (His17, Phe20, and Trp21) the Trp21 is the best binding site for the protonated amino group (−NH_3_^+^) of amantadine derivatives. The cation-π energy (−82.53 kJ/mol) of Trp21 is the largest one among the three aromatic amino acids because of its large π-system. The structural position of Trp21 is also favorable for the binding interaction. The side chain of Trp21 plugs into the p7 channel lumen, which is a good position for the binding interaction, as described in ref [16]: the amino group of amantadine “*on average points to the channel lumen*” (refer to **Fig. 1** and **Fig. 2**). Therefore, from the energetic viewpoint and the structural viewpoint the best binding site for protonated amino group (−NH_3_^+^) of amantadine derivatives is the Trp21.

## Conclusion

In the paper [16] by Chou and colleagues, little attention was given to the pharmacophore amino group of amantadine and rimantadine, so this is now considered in this current manuscript. This study focuses on the recently published NMR structure of HCV p7 and aims to analyze this structure with respect it’s potential binding to adamantine derivatives using molecular modeling and quantum mechanics. Based on the modeling results three potential binding sites (His17, Phe20 and Trp21) are proposed. The best binding site is the Trp21. However, the protonated amino group might change the original binding site to Phe20, which could explain the resistance-conferring Leu20Phe mutation.

All of these residues have been proposed before and have been characterized by mutagenesis, so the data are in line with results from experiments performed in biological systems. Insights are also provided with respect to the know Leu20Phe resistance mutation. The results regarding the binding location of amantadine or rimantadine are confirmatory of the results described in the paper [16] by Chou and colleagues. However, in the current manuscript further results are presented regarding the potential binding of the protonated amino group. We hope that the possible binding sites and the binding interactions, proposed in this study, may help the HCV inhibitor design targeting the p7 channel protein.
